# *catena*-Poly[[diaqua­copper(II)]-μ-hy­drox­ido-κ^2^ *O*:*O*-μ-[4-(4*H*-1,2,4-triazol-4-yl)benzoato]-κ^2^ *N* ^1^:*N* ^2^]

**DOI:** 10.1107/S1600536811032624

**Published:** 2011-08-17

**Authors:** Haochen Shi, Feng Gao, Jingang Qi

**Affiliations:** a College of Physics, Jilin University, Changchun 130012, People’s Republic of China; b Department of Physics Education, Changchun Normal University, Changchun 130032, People’s Republic of China

## Abstract

The title compound, [Cu(C_9_H_6_N_3_O_2_)(OH)(H_2_O)_2_]_*n*_, adopts a chain motif along [010] in which the Cu^II^ atoms are bridged by hy­droxy groups and 4-(1,2,4-triazol-4-yl)benzoate (tab) ligands. The Cu^II^ atom lies on an inversion center and is six-coordinated by two N atoms from two tab ligands, two hy­droxy groups and two water mol­ecules, giving a distorted octa­hedral geometry. The hy­droxy group and the tab ligand are located on a mirror plane. One of the water H atoms is disordered over two positions with equal occupancy factors. Inter­molecular O—H⋯O hydrogen bonds extend the chains into a layer parallel to (100) and C—H⋯O hydrogen bonds connect the layers into a three-dimensional network.

## Related literature

For general background to the applications of coordination polymers, see: Aghabozorg *et al.* (2008 ▶); Liu *et al.* (2010 ▶); Wang *et al.* (2009 ▶); Zhang *et al.* (2004 ▶). For a related structure, see: Lin *et al.* (2011 ▶).
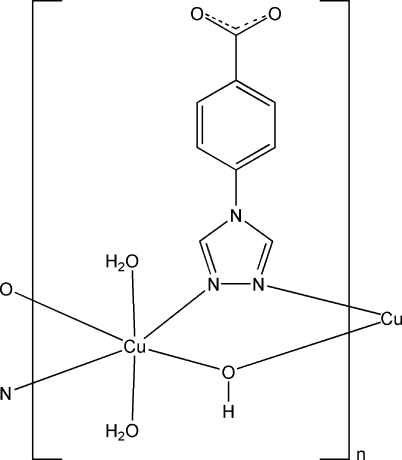


## Experimental

### 

#### Crystal data

[Cu(C_9_H_6_N_3_O_2_)(OH)(H_2_O)_2_]
*M* *_r_* = 304.75
Monoclinic, 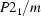

*a* = 6.787 (5) Å
*b* = 6.758 (5) Å
*c* = 12.036 (5) Å
β = 102.919 (5)°
*V* = 538.1 (6) Å^3^
*Z* = 2
Mo *K*α radiation
μ = 2.05 mm^−1^
*T* = 293 K
0.21 × 0.19 × 0.15 mm

#### Data collection

Bruker APEXII CCD diffractometer
Absorption correction: multi-scan (*SADABS*; Bruker, 2001 ▶) *T*_min_ = 0.64, *T*_max_ = 0.75
3021 measured reflections
1165 independent reflections
1010 reflections with *I* > 2σ(*I*)
*R*_int_ = 0.022

#### Refinement

*R*[*F* ^2^ > 2σ(*F* ^2^)] = 0.028
*wR*(*F* ^2^) = 0.083
*S* = 1.12
1165 reflections
111 parameters
4 restraints
H atoms treated by a mixture of independent and constrained refinement
Δρ_max_ = 0.45 e Å^−3^
Δρ_min_ = −0.37 e Å^−3^

### 

Data collection: *APEX2* (Bruker, 2007 ▶); cell refinement: *SAINT* (Bruker, 2007 ▶); data reduction: *SAINT*; program(s) used to solve structure: *SHELXTL* (Sheldrick, 2008 ▶); program(s) used to refine structure: *SHELXTL*; molecular graphics: *SHELXTL*; software used to prepare material for publication: *SHELXTL*.

## Supplementary Material

Crystal structure: contains datablock(s) global, I. DOI: 10.1107/S1600536811032624/hy2457sup1.cif

Structure factors: contains datablock(s) I. DOI: 10.1107/S1600536811032624/hy2457Isup2.hkl

Additional supplementary materials:  crystallographic information; 3D view; checkCIF report

## Figures and Tables

**Table 1:** Hydrogen-bond geometry (Å, °)

*D*—H⋯*A*	*D*—H	H⋯*A*	*D*⋯*A*	*D*—H⋯*A*
O3—H9⋯O2^i^	0.84 (3)	2.07 (3)	2.907 (4)	172 (3)
O4—H10*A*⋯O2^ii^	0.83 (3)	1.94 (3)	2.746 (3)	164 (3)
O4—H10⋯O4^iii^	0.85 (6)	1.94 (6)	2.762 (4)	165 (6)
O4—H10′⋯O4^iv^	0.85 (2)	1.93 (2)	2.759 (4)	165 (7)
C6—H6⋯O1^v^	0.93	2.44	3.172 (5)	135
C8—H8⋯O1^vi^	0.93	2.23	3.052 (4)	147

Symmetry codes: (i) 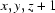; (ii) 
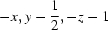
; (iii) 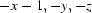; (iv) 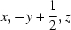; (v) 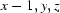; (vi) 
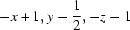
.

**Fig. 1.**
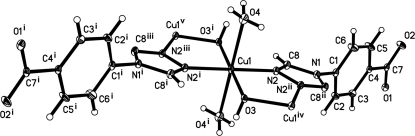
The asymmetric unit of the title compound. Displacement ellipsoids are drawn at the 30% probability level. One H atom of water molecule (O4) is disordered over two positions with equal occupancy factors. [Symmetry codes: (i) -x, -y, -z; (ii) x, 1/2 - y, z; (iii) -x, y - 1/2, -z; (iv) x, y - 1/2, z; (v) x, y + 1/2, z.]

**Fig. 2.**
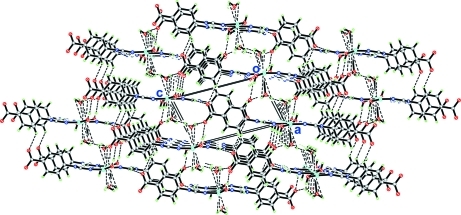
View of the three-dimensional structure of the title compound, built by hydrogen bonds (dashed lines).

## supplementary crystallographic information

### Comment

Coordination polymers are currently of great interest due to structural versatility, unique properties and potential applications in catalysis, gas storage and in molecular-based magnetic materials (Liu *et al.*, 2010; Zhang *et al.*, 2004). Heterocyclic carboxylates have often been used as mono-, bi- or multidentate ligands to bind transition metal centers, leading to the formation of moderately robust metal–organic coordination frameworks (Aghabozorg *et al.*, 2008; Wang *et al.*, 2009). In this contribution, we selected 4-(1,2,4-triazol-4-yl)benzoic acid (Htab) as an organic carboxylate ligand, generating a coordination polymer, [Cu(C_9_H_6_N_3_O_2_)(H_2_O)_2_(OH)], which is reported here.

The title compound adopts a chain motif, in which the hydroxy groups and tab ligands as bridges to connect adjacent octahedrally coordinated Cu^II^ atoms (Fig. 1). The Cu^II^ atom lies on an inversion center and is six-coordinated by two N atoms from two tab ligands, two O atoms from hydroxy groups and two water molecules, giving a distorted octahedral geometry. The Cu—O and Cu—N bond lengths and the O—Cu—O, O—Cu—N and N—Cu—N bond angles are in the normal range (Lin *et al.*, 2011). The hydroxy group and the tab ligand are located on a mirro plane. One of the water H atoms is disordered over two positions with equal occupancy factors. Intermolecular O—H···O hydrogen bonds extend the chains into a layer parallel to (1 0 0). C—H···O hydrogen bonds connect the layers into a three-dimensional network (Fig. 2).

### Experimental

The synthesis was performed under hydrothermal conditions. A mixture of CuCl_2_.2H_2_O (0.2 mmol, 0.034 g), 4-(1,2,4-triazol-4-yl)benzoic acid (0.2 mmol, 0.038 g), NaOH (0.2 mmol, 0.008 g) and H_2_O (15 ml) in a 25 ml stainless steel reactor with a Teflon liner was heated from 293 to 433 K and a constant temperature was maintained at 433 K for 96 h. After the mixture was cooled to 293 K, blue crystals of the title compound were obtained from the reaction.

### Refinement

H atoms on C atoms were positioned geometrically and refined as riding atoms, with C—H = 0.93 Å and *U*_iso_(H) = 1.2*U*_eq_(C). H atoms bonded to O atoms were located in a difference Fourier map and refined with O—H distance restraints of 0.85 (1) Å and with *U*_iso_(H) = 1.5*U*_eq_(O).

### Crystal data

**Table:**

[Cu(C_9_H_6_N_3_O_2_)(OH)(H_2_O)_2_]	*F*(000) = 310
*M**_r_* = 304.75	*D*_x_ = 1.881 Mg m^−^^3^
Monoclinic, *P*2_1_/*m*	Mo *K*α radiation, λ = 0.71073 Å
Hall symbol: -P 2yb	Cell parameters from 1165 reflections
*a* = 6.787 (5) Å	θ = 1.0–26.1°
*b* = 6.758 (5) Å	µ = 2.05 mm^−^^1^
*c* = 12.036 (5) Å	*T* = 293 K
β = 102.919 (5)°	Block, blue
*V* = 538.1 (6) Å^3^	0.21 × 0.19 × 0.15 mm
*Z* = 2	

### Data collection

**Table:**

Bruker APEXII CCD diffractometer	1165 independent reflections
Radiation source: fine-focus sealed tube	1010 reflections with *I* > 2σ(*I*)
graphite	*R*_int_ = 0.022
φ and ω scans	θ_max_ = 26.1°, θ_min_ = 3.1°
Absorption correction: multi-scan (*SADABS*; Bruker, 2001)	*h* = −7→8
*T*_min_ = 0.64, *T*_max_ = 0.75	*k* = −7→8
3021 measured reflections	*l* = −14→12

### Refinement

**Table:**

Refinement on *F*^2^	Primary atom site location: structure-invariant direct methods
Least-squares matrix: full	Secondary atom site location: difference Fourier map
*R*[*F*^2^ > 2σ(*F*^2^)] = 0.028	Hydrogen site location: inferred from neighbouring sites
*wR*(*F*^2^) = 0.083	H atoms treated by a mixture of independent and constrained refinement
*S* = 1.12	*w* = 1/[σ^2^(*F*_o_^2^) + (0.0403*P*)^2^ + 0.5502*P*] where *P* = (*F*_o_^2^ + 2*F*_c_^2^)/3
1165 reflections	(Δ/σ)_max_ < 0.001
111 parameters	Δρ_max_ = 0.45 e Å^−^^3^
4 restraints	Δρ_min_ = −0.37 e Å^−^^3^

### Fractional atomic coordinates and isotropic or equivalent isotropic displacement parameters (Å^2^)

**Table:**

	*x*	*y*	*z*	*U*_iso_*/*U*_eq_	Occ. (<1)
C1	0.2717 (6)	0.2500	−0.3753 (3)	0.0147 (7)	
C2	0.4779 (6)	0.2500	−0.3610 (3)	0.0185 (8)	
H2	0.5611	0.2500	−0.2882	0.022*	
C3	0.5609 (6)	0.2500	−0.4564 (3)	0.0176 (8)	
H3	0.7006	0.2500	−0.4473	0.021*	
C4	0.4374 (5)	0.2500	−0.5653 (3)	0.0135 (7)	
C5	0.2310 (6)	0.2500	−0.5770 (3)	0.0264 (10)	
H5	0.1474	0.2500	−0.6496	0.032*	
C6	0.1448 (6)	0.2500	−0.4827 (3)	0.0279 (10)	
H6	0.0052	0.2500	−0.4915	0.033*	
C7	0.5284 (6)	0.2500	−0.6686 (3)	0.0148 (7)	
C8	0.1359 (4)	0.0900 (4)	−0.2195 (2)	0.0174 (6)	
H8	0.1530	−0.0410	−0.2390	0.021*	
N1	0.1836 (5)	0.2500	−0.2760 (2)	0.0151 (7)	
N2	0.0624 (3)	0.1479 (3)	−0.13349 (17)	0.0150 (5)	
O1	0.7151 (4)	0.2500	−0.6538 (2)	0.0184 (6)	
O2	0.4060 (4)	0.2500	−0.7656 (2)	0.0260 (7)	
Cu1	0.0000	0.0000	0.0000	0.01409 (17)	
O3	0.0096 (4)	0.2500	0.0802 (2)	0.0145 (5)	
O4	−0.3805 (3)	0.0459 (3)	−0.07541 (19)	0.0254 (5)	
H9	0.118 (4)	0.2500	0.130 (3)	0.038*	
H10A	−0.413 (5)	−0.040 (4)	−0.126 (2)	0.038*	
H10	−0.471 (8)	0.017 (11)	−0.040 (6)	0.038*	0.50
H10'	−0.372 (10)	0.169 (2)	−0.086 (6)	0.038*	0.50

### Atomic displacement parameters (Å^2^)

**Table:**

	*U*^11^	*U*^22^	*U*^33^	*U*^12^	*U*^13^	*U*^23^
C1	0.0200 (19)	0.0156 (18)	0.0121 (18)	0.000	0.0112 (15)	0.000
C2	0.0175 (19)	0.026 (2)	0.0116 (18)	0.000	0.0030 (15)	0.000
C3	0.0141 (18)	0.023 (2)	0.0166 (18)	0.000	0.0064 (15)	0.000
C4	0.0189 (18)	0.0116 (17)	0.0118 (18)	0.000	0.0071 (15)	0.000
C5	0.019 (2)	0.053 (3)	0.0076 (18)	0.000	0.0021 (15)	0.000
C6	0.0142 (19)	0.051 (3)	0.020 (2)	0.000	0.0059 (16)	0.000
C7	0.0224 (19)	0.0125 (18)	0.0121 (18)	0.000	0.0092 (15)	0.000
C8	0.0221 (13)	0.0154 (13)	0.0172 (13)	0.0008 (11)	0.0096 (11)	−0.0004 (11)
N1	0.0174 (16)	0.0186 (16)	0.0121 (15)	0.000	0.0090 (12)	0.000
N2	0.0193 (11)	0.0134 (11)	0.0140 (10)	0.0011 (9)	0.0073 (9)	0.0003 (9)
O1	0.0196 (14)	0.0210 (14)	0.0178 (14)	0.000	0.0110 (11)	0.000
O2	0.0230 (15)	0.0456 (19)	0.0098 (13)	0.000	0.0046 (11)	0.000
Cu1	0.0197 (3)	0.0131 (3)	0.0117 (2)	0.00017 (17)	0.00825 (18)	0.00091 (17)
O3	0.0205 (14)	0.0146 (13)	0.0095 (12)	0.000	0.0056 (10)	0.000
O4	0.0300 (11)	0.0221 (11)	0.0270 (11)	−0.0001 (10)	0.0124 (9)	−0.0014 (9)

### Geometric parameters (Å, °)

**Table:**

C1—C2	1.371 (5)	C7—O2	1.272 (4)
C1—C6	1.383 (5)	C8—N2	1.306 (3)
C1—N1	1.452 (4)	C8—N1	1.354 (3)
C2—C3	1.388 (5)	C8—H8	0.9300
C2—H2	0.9300	N2—N2^i^	1.381 (4)
C3—C4	1.389 (5)	Cu1—O3	1.9397 (16)
C3—H3	0.9300	Cu1—N2^ii^	2.016 (2)
C4—C5	1.376 (6)	Cu1—O4	2.558 (3)
C4—C7	1.508 (5)	O3—H9	0.839 (10)
C5—C6	1.388 (5)	O4—H10A	0.836 (10)
C5—H5	0.9300	O4—H10	0.846 (10)
C6—H6	0.9300	O4—H10'	0.844 (10)
C7—O1	1.239 (5)		
			
C2—C1—C6	121.5 (3)	N2—C8—N1	109.6 (2)
C2—C1—N1	119.5 (3)	N2—C8—H8	125.2
C6—C1—N1	119.0 (3)	N1—C8—H8	125.2
C1—C2—C3	119.2 (3)	C8—N1—C8^i^	105.9 (3)
C1—C2—H2	120.4	C8—N1—C1	127.03 (15)
C3—C2—H2	120.4	C8^i^—N1—C1	127.03 (15)
C2—C3—C4	120.6 (3)	C8—N2—N2^i^	107.42 (16)
C2—C3—H3	119.7	C8—N2—Cu1	132.01 (19)
C4—C3—H3	119.7	N2^i^—N2—Cu1	119.72 (6)
C5—C4—C3	118.9 (3)	O3—Cu1—N2	88.58 (10)
C5—C4—C7	120.7 (3)	O3—Cu1—N2^ii^	91.42 (10)
C3—C4—C7	120.4 (3)	N2—Cu1—N2^ii^	180.00 (11)
C4—C5—C6	121.4 (4)	O3—Cu1—O4	89.42 (9)
C4—C5—H5	119.3	O3—Cu1—O4^ii^	90.58 (9)
C6—C5—H5	119.3	N2—Cu1—O4^ii^	88.14 (8)
C1—C6—C5	118.4 (4)	O4—Cu1—N2	91.86 (8)
C1—C6—H6	120.8	Cu1—O3—Cu1^iii^	121.15 (13)
C5—C6—H6	120.8	Cu1—O3—H9	106.6 (15)
O1—C7—O2	124.7 (3)	H10A—O4—H10	96 (5)
O1—C7—C4	118.4 (3)	H10A—O4—H10'	126 (5)
O2—C7—C4	116.9 (3)	H10—O4—H10'	113 (7)
			
N2—C8—N1—C8^i^	0.2 (4)	N1—C8—N2—Cu1	−169.2 (2)
N2—C8—N1—C1	179.1 (3)	C8—N2—Cu1—O3^ii^	−20.5 (3)
C2—C1—N1—C8	−89.3 (3)	N2^i^—N2—Cu1—O3^ii^	171.53 (8)
C6—C1—N1—C8	90.7 (3)	C8—N2—Cu1—O3	159.5 (3)
C2—C1—N1—C8^i^	89.3 (3)	N2^i^—N2—Cu1—O3	−8.47 (8)
C6—C1—N1—C8^i^	−90.7 (3)	N2—Cu1—O3—Cu1^iii^	15.10 (15)
N1—C8—N2—N2^i^	−0.2 (2)	N2^ii^—Cu1—O3—Cu1^iii^	−164.90 (15)

Symmetry codes: (i) *x*, −*y*+1/2, *z*; (ii) −*x*, −*y*, −*z*; (iii) −*x*, *y*+1/2, −*z*.

### Hydrogen-bond geometry (Å, °)

**Table:**

*D*—H···*A*	*D*—H	H···*A*	*D*···*A*	*D*—H···*A*
O3—H9···O2^iv^	0.84 (3)	2.07 (3)	2.907 (4)	172 (3)
O4—H10A···O2^v^	0.83 (3)	1.94 (3)	2.746 (3)	164 (3)
O4—H10···O4^vi^	0.85 (6)	1.94 (6)	2.762 (4)	165 (6)
O4—H10'···O4^i^	0.85 (2)	1.93 (2)	2.759 (4)	165 (7)
C6—H6···O1^vii^	0.93	2.44	3.172 (5)	135
C8—H8···O1^viii^	0.93	2.23	3.052 (4)	147

Symmetry codes: (iv) *x*, *y*, *z*+1; (v) −*x*, *y*−1/2, −*z*−1; (vi) −*x*−1, −*y*, −*z*; (i) *x*, −*y*+1/2, *z*; (vii) *x*−1, *y*, *z*; (viii) −*x*+1, *y*−1/2, −*z*−1.
